# Crack Failure Analysis of Hot-Stamping Die Insert for Manufacturing an Automobile A-Pillar

**DOI:** 10.3390/ma18133052

**Published:** 2025-06-27

**Authors:** Shuo Wang, Zhiyang Dou, Yixiu Yin, Hanqi Zhao, Yaocheng Wang, Pengpeng Zuo, Na Min, Senlin Jin

**Affiliations:** 1 School of Materials Science and Engineering, Jiangsu University of Science and Technology, Zhenjiang 212003, China; 222210605223@stu.just.edu.cn (S.W.); zydou0126@163.com (Z.D.); 19707206802@163.com (Y.Y.); qibcc2496iky2@163.com (H.Z.); w13558649531@163.com (Y.W.); 2 Zhejiang Qingshan Iron and Steel Co., Ltd., Lishui 323903, China; 3 School of Materials Science and Physics, University of Mining and Technology, Xuzhou 221116, China; 4 School of Materials Science and Engineering, Shanghai University, Shanghai 200072, China; minnacy@shu.edu.cn; 5 National Industry Metrology and Measurement Center of Graphene Material (Shenzhen), Shenzhen Institute for Technology, National Institute of Metrology (NIM), Shenzhen 518107, China

**Keywords:** hot-stamping die, crack failure, cooling channel, stress-induced corrosion

## Abstract

In order to determine the failure reason for the non-working area of a cracked A-pillar hot-stamping die insert, various instruments were used to detect the properties and microstructures of the cracks and matrix. The results show that the cracks are located in the area where the oxidative corrosion is more serious, and the cracks do not appear in the pitting area, verifying that crack initiation is related to the stress concentration on the upper half of the inner wall of the cooling channel. Meanwhile, pores and cracks exist in the grain boundary and crystal, making the impact energy of the die steel poor. Therefore, crack initiation and propagation easily occur along the brittle oxide layer. In summary, the die insert is damaged by stress-induced corrosion. In engineering applications of hot-stamping dies, we should pay more attention to the cracking of the cooling channel caused by stress and corrosion.

## 1. Introduction

During the past decade, international automobile exhaust emission standards are becoming increasingly strict, and the battery life of new energy vehicles needs to be improved [1,2]. A lightweight car body has become the main theme of the automobile industry [3]. For this purpose, major companies involved in auto parts and components manufacturing have achieved the goal of a lightweight car body by using a hot-stamping process for advanced high-strength steel sheets, which does not reduce the safety performance of automobiles [4,5,6]. With the popularization of the hot-stamping process, auto parts and components have become more and more complex, posing new challenges to the performance of hot-working dies [7,8].

In the hot-stamping process, the die must withstand the stress caused by mechanical loading [9], metal-plastic flow [10], and thermal fatigue of cycling at 200–400 °C [11]. Therefore, most researchers believe that the mechanical stress and the thermal stress accumulate continuously during tens of thousands of stamping processes (the service life of the hot-stamping die is about 200,000 cycles), causing damage and cracking of the die [12,13]. Wang and Wu [14] found that the precipitation of carbides along the grain boundary weakens its binding force, which is prone to crack under thermal mechanical stress. Zhang et al. [15] also revealed that the grain boundary is closely related to the life of the die steel. In experiments, dislocations can cross a small angle grain boundary and slip from one grain to the adjacent grain. However, dislocations cannot cross the high-angle grain boundary, causing a large amount of dislocation to accumulate at the grain boundary, which increases the stress at the grain boundary. Attila et al. [16] point out that stress corrosion occurs in the die during service, accelerating the crack propagation at the grain boundary. Valls et al. [17] observed an oxide layer in the cooling channel of the hot-stamping die insert, which is favorable for stress corrosion cracking.

In the actual engineering application of hot-stamping dies, in addition to normal wear failure, according to incomplete statistics, more than 90% of failed dies start to crack from the inner walls of the water channels and then extend to the working surface, causing water leakage and rendering them unusable. Therefore, as this situation frequently occurs in engineering, it is necessary to further analyze its root cause. Despite a lot of research on the cause of die failure, these studies do not provide much attention to the cracks on the non-working area of the die insert. For this reason, this paper aims to emphasize the effects of stress and corrosion on the insert by analyzing the abnormal cracking of the non-working area of the die insert.

## 2. Background and Experimental Information

**Figure 1:**
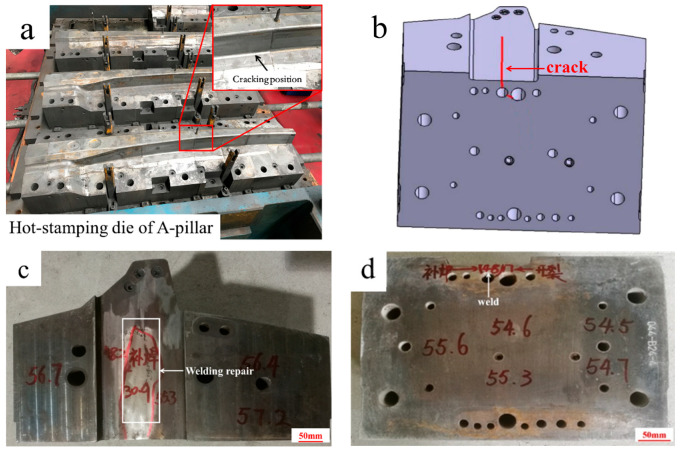
Photos of the cracked hot-stamping die insert of the side beam reinforcement sheet for an automobile A-pillar: (**a**) physical photo of an A-pillar hot-stamping die; (**b**) design diagram of the cracked insert; (**c**) physical photo of the cracked insert and photo of the HRC test results near the weld area; (**d**) photo of the HRC test results of the non-welded area.

**Figure 2:**
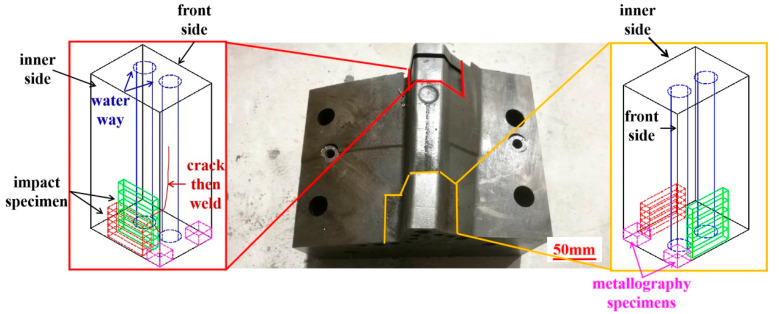
Schematic diagram of the samples cut from a cracked hot-stamping die insert.

Figure 1a shows the cracked hot-stamping die for manufacturing the side beam reinforcement sheet for an automobile A-pillar. In die service, the insert is subjected to cyclic impact loads and thermal load cycling ranging from 200 to 400 °C. After 50,000 cycles (25% of the normal service life), the cracks occur at the cooling channel bottom where the non-working area is located. As shown in Figure 1b,c, the cooling channel below the die insert cavity is blocked, so the cooling water is passed from the bottom of the cooling channel. The schematic diagram of the metallography sample and the impact sample is shown in Figure 2.

The chemical composition of the die insert was determined using a spectrometer (QSG 750. II, OBLF, Witten, Germany). According to GB/T 229-2020 [18], the impact tester (ZBC2602-CE, MTS, Shanghai, China) was used to measure the unnotched impact energy of the die steel. The microstructure of the die steel was observed using an optical microscope (OM, MA100, Nikon, Tokyo, Japan) and a scanning electron microscope (SEM, Zeiss, Supra 40, Oberkochen, Germany). The impact fracture morphology was observed using a scanning electron microscope (SEM, Zeiss, Supra 40, Germany). The inner wall morphology of the cooling channel was observed using a stereomicroscope (VHX-600, Keyence, Osaka, Japan).

## 3. Results

### 3.1. Chemical Composition

The chemical composition is given in Table 1. Obviously, the cracked die insert was 4Cr5Mo2V steel, which is a universal hot-working die steel with excellent toughness with a Rockwell hardness (HRC) of 52.0–56.0. When the HRC is 52.0–54.0, the tensile strength of 4Cr5Mo2V steel is 1880 MPa, and the 0.2% yield strength is 1560 MPa.

### 3.2. Surface Hardness

The surface hardness of the die insert was measured using the Leeb hardness (HL) tester (Equotip 550, Proceq, Schwerzenbach, Switzerland), and then the HRC value was obtained with conversion calculations. The results are shown in Figure 1c,d. The surface hardness of the die insert is uniform within HRC 54.0–56.0, but the hardness of the position near the weld is low within HRC 30.0–32.0. The results show that the surface hardness of the die insert is slightly increased. The die insert has no nitrogen based on querying the heat treatment record.

### 3.3. Non-Metallic Inclusion

**Figure 3:**
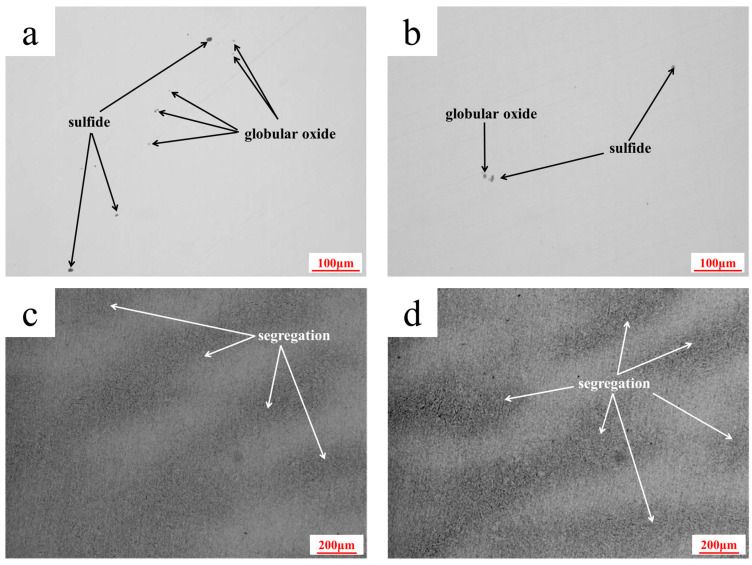
OM micrographs of the die steel: (**a**) non-metallic inclusions in the area near the weld; (**b**) non-metallic inclusions in the non-welded area; (**c**) banding segregation in the area near the weld; (**d**) banding segregation in the non-welded area.

In accordance with NADCA #207-2016 standard [19], the metallography method is used to evaluate the non-metallic inclusions of the die steel. The micrographs of the non-metallic inclusions are presented in Figure 3a,b. The level results are shown in Table 2. The inclusions are similar in the area near the weld and non-welded area of the die insert. The die steel contains a small amount of sulfide and globular oxides. The microcleanliness is excellent and fully meets the requirements of the hot-stamping die steel.

### 3.4. Microstructure Characterization

In this paper, the banding segregation of the die steel is evaluated according to the NADCA #207-2016 standard [19]. The OM microstructure is shown in Figure 3c,d. It can be observed that the banding segregation at the area near the weld and the non-welded area are at the same level. Eutectic carbide was not observed in each of the segregation bands, and the banding segregation level was “Acceptable” based on conformance with the NADCA # 207-2016 banding segregation reference chart for the levels of microbanding [19].

### 3.5. Crack Observation

**Figure 4:**
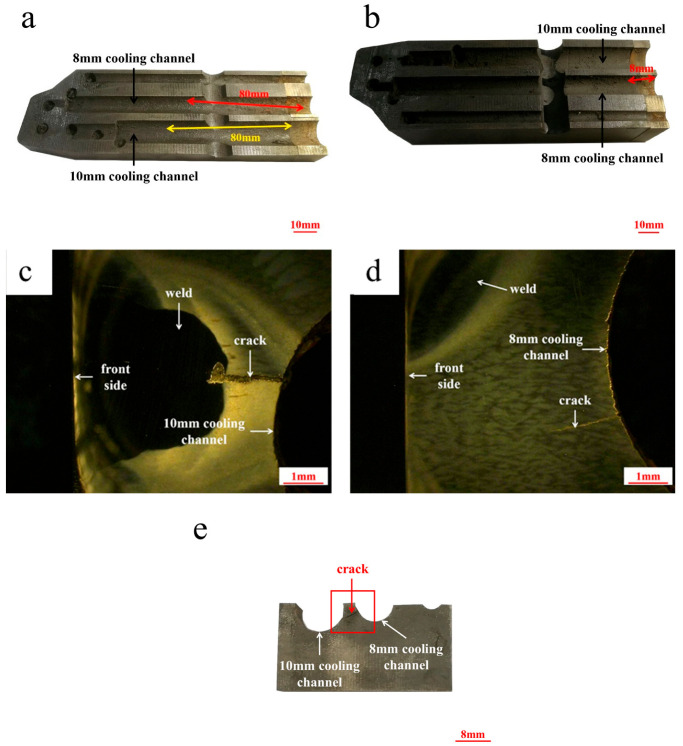
Photos of cracks on the cross-section of the cooling channel: (**a**,**c**,**d**) the upper half; (**b**) and (**e**) the lower half.

The cross-section photo of the cooling channel is shown in Figure 4. Three cracks can be observed in the field of the die insert. The crack propagation direction is consistent with the direction of the cooling channel. Two of the cracks are in the upper half part of the cooling channel, both lengths of which are about 80 mm, seen in Figure 4a. Another crack is located in the lower half of the cooling channel, and the length is about 8 mm, as seen in Figure 4b. The results point out that the upper half of the cooling channel is more conducive to crack propagation than the lower half part of cooling channel. The crack in the cooling channel with a diameter of 10 mm was initiated in the inner wall of the cooling channel and stopped near the weld position (Figure 4c). The crack of the lower half part of the cooling channel with a diameter of 8 mm was initiated in the inner wall of the cooling channel (Figure 4d) and extended to another cooling channel with a diameter of 10 mm (Figure 4e). It indicates that the cooling channel is favorable for the initiation of cracks.

**Figure 5:**
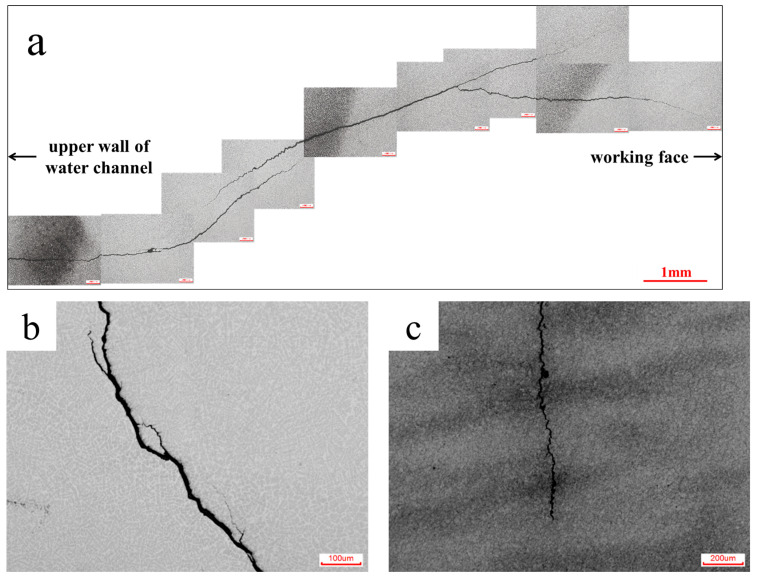
Typical crack morphology: (**a**) complete morphology of a crack originating from the upper wall of the water channel to the working surface; (**b**) characteristics of intergranular and transgranular cracks; (**c**) the mapping relationship between cracks and banded segregation.

As can be seen from Figure 4, the crack originated from the upper wall of the water channel and then expanded perpendicular to the pressing direction until it reached the working surface. The typical crack morphology can be also seen in Figure 5. Figure 5a shows the complete morphology of the cracks that originated from the upper wall of the water channel to the working surface. Figure 5b shows crack propagation that occurs in a mixed form of intergranular and transgranular propagation. This indicates that mixed plastic and brittle cracks are observed more often in the fracture of hot-stamping die steels. Figure 5c shows the mapping relationship between cracks and banded segregation. It can be seen that the cracks generally extend perpendicular to banded segregation. This suggests that the selected die working surface is parallel to the hot-working elongation direction of the steel bloom, which is a common standard in die manufacturing.

**Figure 6:**
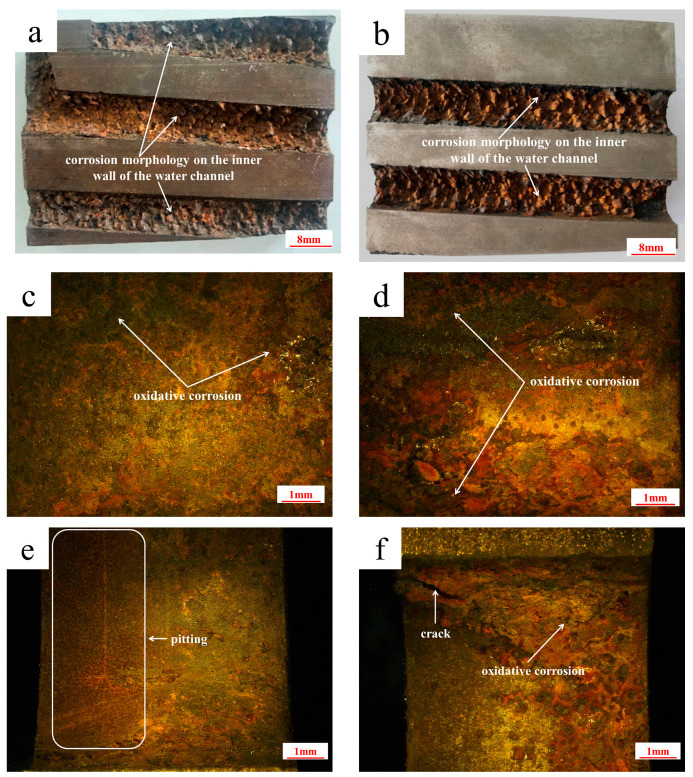
Corrosion morphology on the inner walls of the water channel: (**a**,**b**) the corrosion morphology on the inner walls of the *ϕ* 8 mm water channel; (**c**,**d**) the upper half; (**e**,**f**) the lower half.

The corrosion morphology on the inner walls of the water channel with a diameter of 8 mm is shown in Figure 6a,b. The upper (Figure 6c,d) and lower (Figure 6e,f) half of the inner wall in the cooling channel are both corroded, but the upper half is more severely corroded than the lower half. There is a large amount of red-brown iron oxide. When the localized iron oxides fall off, the matrix with less corrosiveness is exposed. Therefore, obvious undulations can be discovered on the surface of the cooling channel. At the same time, cracks exist in the area where the water channel is severely corroded, as shown in Figure 6d.

**Figure 7:**
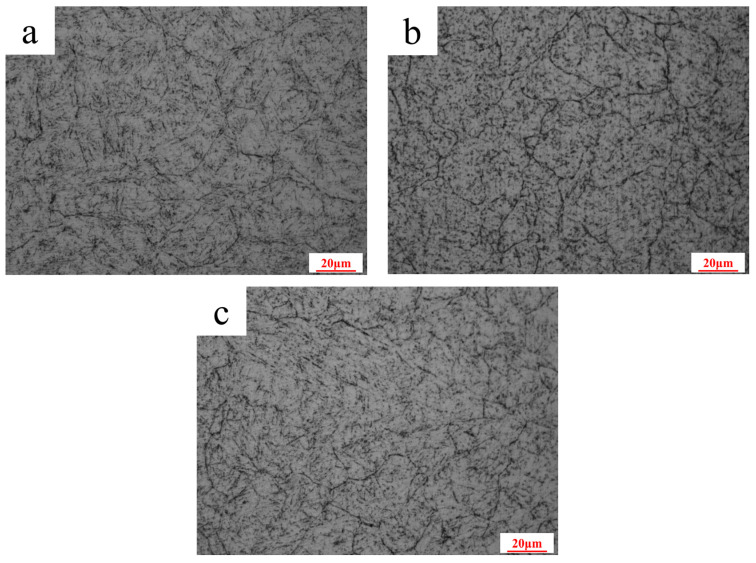
OM microstructure graphs: (**a**) the upper half of the area near the weld; (**b**) the lower half of the area near the weld; (**c**) non-welded area.

After mechanical grinding and polishing, the OM microstructures of the inner wall in the cooling channel, the area near the weld, and the non-welded area are shown in Figure 7. It is difficult to observe the sub-twisted microstructure in tempered martensite after hot-stamping cycling ranging from 200 to 400 °C. The microstructure is relatively uniform, and the grain boundary is more obvious. There are black round or elliptical particles in the crystal and the grain boundary.

### 3.6. Impact Toughness

The non-notch impact energy at different positions with different directions was tested. The impact samples were processed according to the NADCA#207-90 standard [20]. The sample size is 7 mm × 10 mm × 55 mm, and the results of the impact experiment are given in Table 3. Since the impact sample of the die insert without a weld (the impact direction is perpendicular to the cooling channel) is closer to the core of the die insert, the average impact energy is higher (245 J). The average impact energy values of other locations are generally low (37–96 J), which is only 15–39% of the matrix material. There is a significant difference in the impact energy near the cooling channel of the die insert and the core region of the die insert. Due to the similar grades of the inclusion and banding segregation in different regions, the low impact energy is not affected by the quality of the die steel, but it is affected by stress-induced corrosion behavior in the cooling channel.

**Figure 8:**
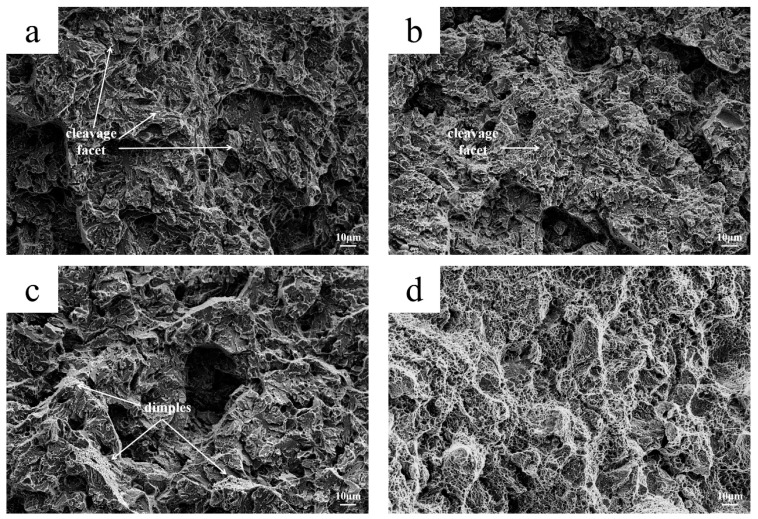
SEM graphs of impact fracture: (**a**) 37 J; (**b**) 43 J; (**c**) 106 J; (**d**) 325 J.

The fracture surface of the impact specimens with impact energies of 37 J, 43 J, 106 J, and 325 J were observed using SEM. The fracture micrographs are shown in Figure 8. There are obvious cleavage steps and a few intergranular features on the 37 J and 43 J impact specimens, as shown in Figure 8a,b, which is a typical brittle intergranular and transgranular mixed cleavage fracture. In Figure 8c, the dimple characteristics can be observed in the 106 J impact specimen. There is no crystal structure in the fracture of the 325 J impact specimen, and all the cross-sections exhibit fiber characteristics. Meanwhile, there are many fine dimples, which are microporous aggregate fractures.

**Figure 9:**
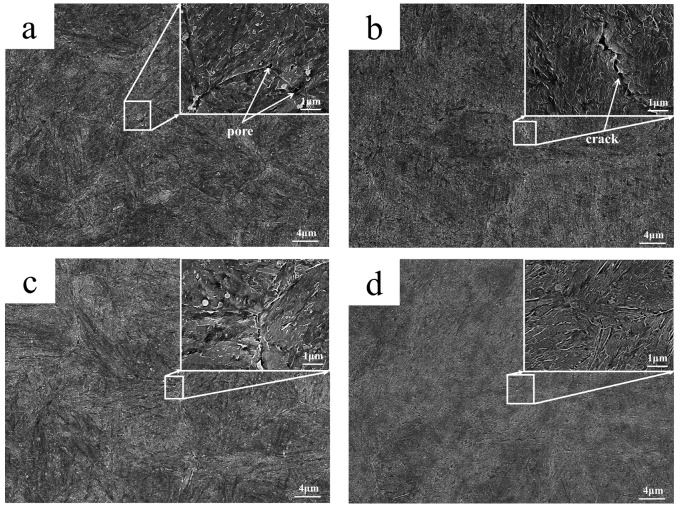
SEM microstructures of impact specimens: (**a**) 37 J; (**b**) 43 J; (**c**) 106 J; (**d**) 325 J.

**Figure 10:**
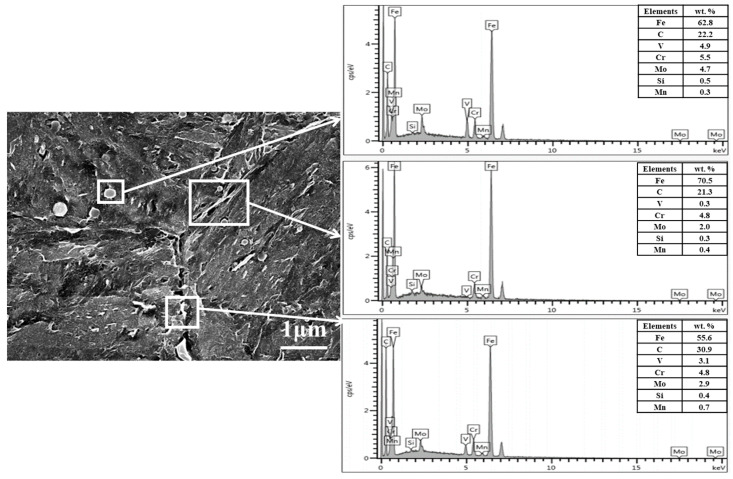
EDS analysis of carbides at grain boundaries in the 106 J impact sample.

The grain boundary SEM graphs of the 37 J, 43 J, 106 J, and 325 J impact samples are as shown in Figure 9. The tempered martensite morphology of all the samples has disappeared, and the carbides are fine and evenly distributed. At the same time, there are pores and cracks on the intragranular and grain boundary of the 37 J, 43 J, and 106 J impact specimens. The pores and cracks are 0.5–2.0 μm in size in the 37 J and 43 J impact samples, as shown in Figure 9a,b. In addition, carbides of similar sizes precipitate at the grain boundaries and coarsen, as shown in Figure 9c,d. That is, there is a certain degree of grain boundary damage at the grain boundaries. EDS analysis of the grain boundary precipitation phase is shown in Figure 10. It can be seen that the steel is rich in C and alloying elements such as Cr, Mo, and V. The grain boundaries exhibit widening and damage, and some carbides have coarsened. Therefore, it can be inferred that as hot-stamping proceeds, carbides gradually precipitate at grain boundaries and some carbide coarsening also occurs. This is the reason for the low impact energy of the steel and its brittle behavior. This point can be confirmed by studying thermal mechanical fatigue simulations of hot-stamping service conditions. Hofinger et al. [21] found that second-phase precipitation and coarsening occur in hot-work die steel during thermomechanical fatigue.

The black particles on the grain boundary are pores and cracks. The 325 J impact sample has higher grain boundary energy and a smaller number of pores on the grain boundary. In summary, the more pores there are on the intragranular areas and small cracks on the grain boundary, the lower the impact energy of the sample. Thus, the impact toughness is related to the number of pores on the intragranular areas and small cracks on the grain boundary.

## 4. Discussion

From Figure 3 and Table 3, the die steel exhibits good metallurgical quality, microstructure, and impact toughness, which meet the NADCA#207-2016 standard [19] requirements. Thus, the material itself is not the key cause of the cracked die insert. In Figure 4 and Figure 6, the corrosion of the cooling channel is obvious. The cracks in the die insert sprout and spread along the cooling channel. All the preliminary results point out that there is stress-induced corrosion in the cooling channel. The stress-induced corrosion is favorable for the initiation and expansion of the cracks.

**Figure 11:**
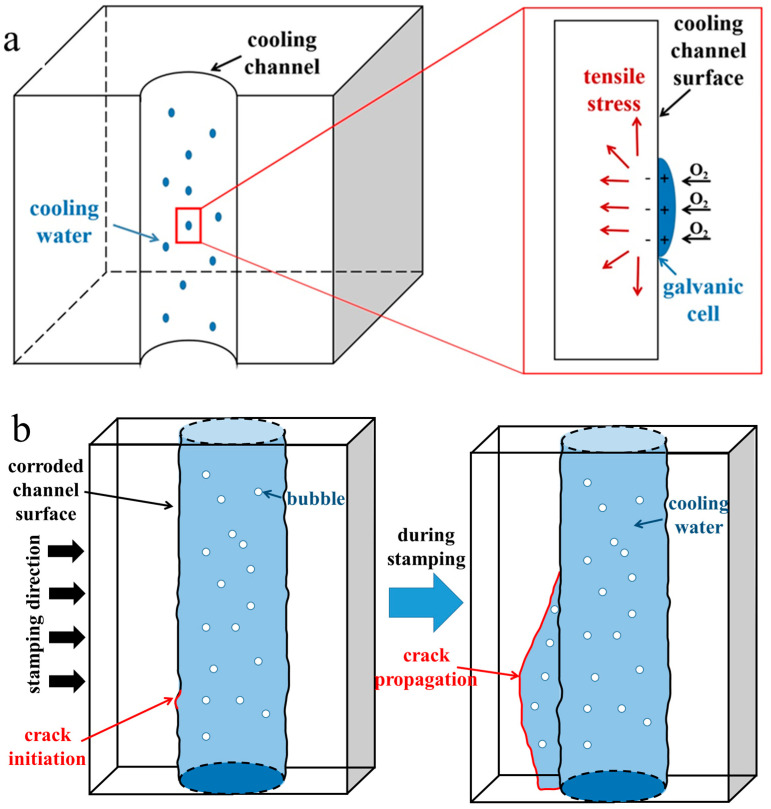
Schematic diagram of the mechanism: (**a**) stress-induced corrosion on the cooling channel; (**b**) crack propagation on the cooling channel during hot-stamping.

Usually, during the service, the die insert is filled with deionized water to achieve the high strength of the hot-stamping steel sheet by quenching in the die [22]. Because the gas content in the cooling channel was low during hot-stamping, the pitting and oxidative corrosion will not appear under 50,000 cycles during the service process. When viewing the service record, it was found that the die insert was offline for about one month. The protection of the cooling channel was not provided before the die was offline, resulting in high humidity in the cooling channel. During the hot-stamping process, the addition of mechanical stress will increase the residual tensile stress. In Figure 11a, when water vapor condenses on the surface of the cooling channel, the residual tensile stress promotes electron exchange between oxygen and iron atoms to form a galvanic cell, which promotes oxidative corrosion of the cooling channel.

However, the cause of stress-induced corrosion cracks is controversial [23]. Some researchers believe that the large stress concentration is the primary factor for stress corrosion cracking on smooth surfaces [24]. Other researchers have also found that under corrosive conditions, inclusions will form pitting pits, and plastic strain zones exist at the bottom of the pit, which provides favorable conditions for the initiation of stress corrosion cracking [25,26]. In this paper, the crack is in the area where the oxidative corrosion is more serious, and the crack does not appear in the pitting area, verifying that the crack initiation is related to the stress concentration. As shown in Figure 4a,d, the crack with a length of 80 mm on the inner wall of the *ϕ* 8 mm cooling channel propagates along the direction of water flow. Observing the cross-section of the cooling channel, the crack depth is only 1.5 mm, and the crack does not extend to the surface of the die insert. It indicates that the crack is easy to spread along the cooling channel. In Figure 11b, during the stamping process, the die insert is subjected to compressive stress, and the maximum stress direction is perpendicular to the stamping process. Thus, the crack should be easily extended perpendicular to the cooling channel. However, it was found that an oxide layer exists on the surface of the cooling channel, and the crack is more likely to expand in the direction of the cooling channel. Since the oxide is a hard and brittle phase, it is easy to rupture upon withstanding external stress during hot-stamping. Then, the cracks originate and expand in the subsequent service. When cooling water has a high oxygen content, the crack tip consumes a large amount of oxygen, but the replenishment velocity is low. In this case, the potential of the tip surface is low and favorable for the gathering of impure elements at the crack tip. It can hinder the passive surface and promote anodic dissolution, finally causing the crack to expand [27].

**Figure 12:**
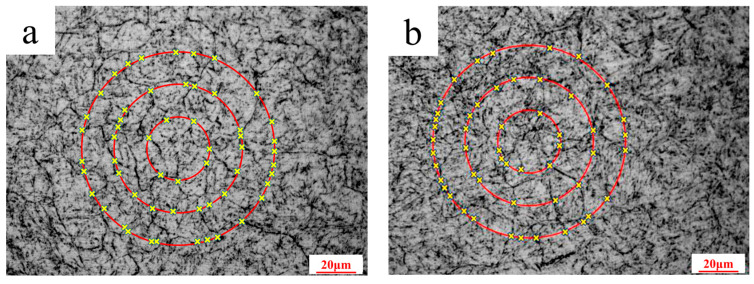
Grain size rating photos: (**a**) 37 J; (**b**) 43 J.

It can be seen from Table 3 that the average impact energy of the non-cracked part (impact direction perpendicular to the cooling channel) is higher, and the highest impact energy is 325 J. In contrast, the average impact energies of the other three sets of impact samples are abnormally low. As shown in Figure 8, the cleavage planes of the fractures of the 37 J and 43 J specimens were found to be wider. The fracture does not show any ductility, indicating that the atomic combination in the crystal is weak. Only uniform and small dimples can be observed in the fracture of the 325 J sample, showing that the atomic combination in the crystal is strong. It can be seen from Figure 9 that the grain boundary weakening phenomenon exists in the 37 J, 43 J, and 106 J impact samples. Black particles with a size of about 0.5–2.0 μm exist in both the grain boundary position and the substrate position. It was confirmed by SEM observation that the black particles are intracrystalline pores and grain boundary cracks. The carbides and matrix microstructure or the grain boundary of the 325 J impact specimen are tightly combined, while the pores between the carbides and the matrix exists in the 37 J, 43 J, and 106 J impact specimens, as shown in Figure 9. Due to the high rigidity of the carbide, there is uneven deformation between the carbide and the matrix under cyclic stress; thus, the mismatch of the interface increases. On the other hand, under stress conditions, many dislocations move along the sliding surface toward the grain boundary. Grain boundary dislocations induce a stress concentration and gradually form voids. The grain sizes of the 37 J and 43 J impact samples are both level 10, as measured by the ternary intercept method, as shown in Figure 12. The austenitizing grain size of 4Cr5Mo2V is level 8.0–8.5, indicating that the intragranular dislocations accumulate in different regions and induce a stress concentration. It gradually transforms from the small angular grain boundary to large angular grain boundary, which divides and refines crystal grains [28].

Dislocation proliferation requires thermal stress or mechanical stress. The stress of the failure die insert is mainly related to the pressure load. In the single hot-stamping process, the tensile stress of the die is much lower than the yield strength of the steel. However, in the cyclic hot-stamping process, the stress continuously accumulates, and the tensile stress in the steel continuously increases. The combination of thermal stress and mechanical stress made the period required for stress-induced corrosion cracking shorter. The number of dislocations and defects in the cooling channel provides favorable conditions for oxidative corrosion and promotes crack initiation and expansion.

## 5. Conclusions

The main reasons for the crack failure of the non-working area are twofold: (1) The cooling channel of the die insert is not effectively protected, leading to the cooling channel being corroded seriously. (2) During the service of 50,000–80,000 cycles, the dislocation density of the die steel was not reduced by stress relief annealing.

Regarding the cause of die failure, four precautions are proposed: (1) The cooling channel should be cleaned with a weak acid solvent or high-pressure water after the die is offline. (2) The cooling channel should be air-dried with compressed air, and the oil-based protective layer should be coated to reduce the corrosion risk of the cooling channel. (3) The die should be subjected to stress relief annealing to reduce the dislocation density of the die steel. (4) The phenomenon of excessive stress in a certain area should be avoided according to the die design.

## Figures and Tables

**Table 1:** Chemical composition of cracked die steel (mass fraction, %).

Element	C	Si	Mn	V	Mo	Cr	P	S	Fe
Content	0.39	0.22	0.43	0.63	2.34	5.00	0.010	0.002	Bal.

**Table 2:** Level of non-metallic inclusions in the die steel.

Inclusion Type	Grade A		Grade B		Grade C		Grade D	
	Fine	Heavy	Fine	Heavy	Fine	Heavy	Fine	Heavy
Levels in NADCA#207-2016	0.5	0.5	1.0	1.0	0.5	0.5	1.0	1.0
Near the weld	0.5	0.5	0.5	0.5	0.5	0.5	1.0	0.5
Non-welded	0.5	0.5	0.5	0.5	0.5	0.5	1.0	0.5

**Table 3:** Impact energy of the die steel at different positions.

Position	Impact Energy, J					
	No. 1	No. 2	No. 3	No. 4	No. 5	Average Value
Near the weld area (impact direction parallel to the cooling channel)	45	43	37	20	40	37
Near the weld area (impact direction perpendicular to the cooling channel)	84	106	85	104	100	96
Non-welded area (impact direction parallel to cooling channel)	71	45	43	89	64	62
Non-welded area (impact direction is perpendicular to the cooling channel)	265	325	167	270	200	245

## Data Availability

The original contributions presented in this study are included in the article; further inquiries can be directed to the corresponding authors.

## References

[1] Jin X., Gong Y., Han X., Du H., Ding W., Zhang Y., Feng Y., Ma M., Liang B., Zhao Y. (2020). A review of current state and prospect of the manufacturing and application of advanced hot stamping automobile steels. Acta Metall. Sin..

[2] Costa L.D.L., Brito A.M.G., Rosiak A., Schaeffer L. (2020). Study of the applicability of 22MnB5 sheet metal as protective masks to improve tool life in hot forging process. Int. J. Adv. Manufact. Technol..

[3] Jiang B., Li X., Zuo P., Wu X. (2020). Study on isothermal fatigue life prediction model of a new type hot stamping die steel 4Cr2Mo2V. Eng. Fail. Anal..

[4] Escher C., Wilzer J. (2015). Tool steels for hot-stamping of high strength automotive body parts. Int. Conf. Stone Concr. Mach. (ICSCM).

[5] Tisza M., Imre C. (2018). Comparative study of the application of steels and aluminum in lightweight production of automotive parts. Int. J. Lightweight Mater. Manuf..

[6] Abdullah N.A.Z., Sani M.S.M., Salwani M.S., Husain N.A. (2020). A review on crashworthiness studies of crash box structure. Thin-Walled Struct..

[7] Shi D., Watanabe K., Naito J., Funada K., Yasui K. (2022). Design optimization and application of hot-stamped B pillar with local patchwork blanks. Thin-Walled Struct..

[8] Lu R., Gao W., Hu X., Liu W., Li Y., Liu X. (2018). Crushing analysis and crashworthiness optimization of tailor rolled tubes with variation of thickness and material properties. Int. J. Mech. Sci..

[9] Li S., Wu X., Li X., Li J., He X. (2016). Wear characteristics of Mo-W-type hot-work steel at high temperature. Tribol. Lett..

[10] Barrau O., Boher C., Gras R., Rezai-Aria F. (2003). Analysis of the friction and wear behaviour of hot work tool steel for forging. Wear.

[11] Song X., Zhan P. (2017). Study on temperature field of hot-stamping dies for b pillar of vehicles. CFHI Technol..

[12] Bernhart G., Moulinier G., Brucelle O., Delagnes D. (1999). High temperature low cycle fatigue behaviour of a martensitic forging tool steel. Int. J. Fatigue.

[13] Jung I., Lubich V., Wieland H.-J. Tool failure–causes and prevention. Proceedings of the 6th International Tooling Conference.

[14] Wang W., Wu X. (2017). Analyses on cracking reasons of a hot-stamping B-pillar die insertges. Die Mould Manuf..

[15] Zhang Z., Wang Z., Shi C. (2004). Intergranular fatigue cracking mechanisms. J. Grad. Sch. Chin. Acad. Sci..

[16] Alkan A., Gümüş S., Atapek Ş.H., Polat Ş. (2016). A case study of a stress corrosion cracking failure in an AA5083 mold material used for curing rubber compounds. Prot. Met. Phys. Chem. Surf..

[17] Valls I., Casas B., Rodríguez N., Paar U. (2010). Benefits from using high thermal conductivity tool steels in the hot forming of steels. La Metall. Ital..

[18] (2000). Metallic Materials—Charpy Pendulum Impact Test Method.

[19] (2016). Special Quality Die Steel & Heat Yreatment Acceptance Criteria For Die Casting Dies.

[20] (1990). Premium Quality H13 Steel Acceptance Criteria For Pressure Die Casting Dies.

[21] Hofinger M., Seisenbacher B., Ognianov M., Leitner H., Turk C., Kapp M., Schnitzer R. (2020). Thermomechanical fatigue testing of dual hardening tool steels. Steel Res. Int..

[22] Mori K.I., Bariani P.F., Behrens B.A., Brosius A., Bruschi S., Maeno T., Merklein M., Yanagimoto J.J.C.A. (2017). Hot stamping of ultra-high strength steel parts. CIRP Ann..

[23] Elboujdaini M., Revie R. (2009). Metallurgical factors in stress corrosion cracking (SCC) and hydrogen-induced cracking (HIC). J. Solid State Electr..

[24] Parkins R.N., O′Dell C.S., Fessler R.R. (1984). Factors affecting the potential of galvanostatically polarised pipeline steel in relation to scc in co2-3-hco-3 solutions. Corros. Sci..

[25] Wang L., Xin J., Cheng L., Zhao K., Sum B., Li J., Wang X., Cui Z. (2019). Influence of inclusions on initiation of pitting corrosion and stress corrosion cracking of X70 steel in near-neutral pH environment. Corros. Sci..

[26] Turnbull A., Wright L., Crocker L. (2010). New insight into the pit-to-crack transition from finite element analysis of the stress and strain distribution around a corrosion pit. Corros. Sci..

[27] Peng Q., Li G., Shoji T. (2003). The crack tip solution chemistry in sensitized stainless steel in simulated boiling water reactor water studied using a microsampling technique. J. Nucl. Sci. Technol..

[28] Valiev R. (2004). Nanostructuring of metals by severe plastic deformation for advanced properties. Nat. Mater..

